# The Impact of Herbal Additives for Poultry Feed on the Fatty Acid Profile of Meat

**DOI:** 10.3390/ani12091054

**Published:** 2022-04-19

**Authors:** Karolina Jachimowicz, Anna Winiarska-Mieczan, Ewa Tomaszewska

**Affiliations:** 1 Institute of Animal Nutrition and Bromatology, University of Life Sciences in Lublin, Akademicka St. 13, 20-950 Lublin, Poland; anna.mieczan@up.lublin.pl; 2 Department of Animal Physiology, Faculty of Veterinary Medicine, University of Life Sciences in Lublin, Akademicka St. 12, 20-950 Lublin, Poland; ewarst@interia.pl

**Keywords:** herbs, herbal additives, fatty acid profile, broiler chickens, meat quality

## Abstract

**Simple Summary:**

Certain herbs, including their properly selected mixtures or preparations, can have a positive influence on the animal body. Works discussing the impact of herbs on poultry meat quality have gained increasing popularity. This paper aims to review reference literature on the influence of herbal additives for broiler chicken diets on the fatty acid profile of poultry meat. Fat plays a key role both as regards the growth of animals and the nutritional value and quality of meat products. Poultry products are vastly popular, so enriching favorite meat with sources of unsaturated fatty acids is a good way of improving the health of the population.

**Abstract:**

Researchers often found that herbal additives to chicken feed can favorably alter the fatty acid profile of the meat. The most desirable effects of diet modification comprise an increased content of polyunsaturated fatty acids (PUFA) and monounsaturated fatty acids (MUFA) and a reduced content of saturated fatty acids (SFA) in the breast and thigh muscles. A modified fatty acid profile contributes to improvement in the quality of poultry meat, which is reflected in its increased consumption. However, it may be problematic that PUFAs are oxidized easier than other lipids, which can have a negative impact on the sensory traits of meat. By contrast, herbs and herbal products contain antioxidants that can prevent the oxidation of unsaturated fatty acids and cholesterol present in animal-origin products and increase the antioxidant potential of the consumer’s body. This paper aims to review the influence of herbal additives for broiler chicken diets on the fatty acid profile of poultry meat. Special attention was paid to changes in the content of SFAs, MUFAs, and PUFAs, but also alterations in the omega-6:omega-3 ratio. The presented reference literature supports the statement that herbs and bioactive components of herbs added to chicken diets can improve the quality of broiler chicken meat by altering the content of fatty acids.

## 1. Introduction

Over the past decade, both the well-being of birds and food safety were the priorities for poultry producers. Under the pressure of consumers, the use of artificial feed additives is abandoned, and extreme solutions related to intensive rearing are mitigated to ensure the protection of the environment. Many authors aim to identify herbs that affect both the fatty acid profile and the antioxidant status [1,2,3]. The quality of poultry meat is an essential factor influencing consumer health [4]. Breast meat can be regarded as an important component of a healthy diet [5] as it contains more polyunsaturated fatty acids (PUFA) and less saturated fatty acids (SFA) than meat from other species of animals, e.g., beef and lamb [6]. Furthermore, its global consumption is growing [7] as it perfectly fits modern culinary trends where easy and fast cooking is preferred. Phytobiotics, preparations of plant origin derived from herbs that contain bioactive secondary metabolites, can be an interesting feed additive alternative to various growth stimulants, traditionally used in animal nutrition [8]. The wide spectrum of effects of such additives includes improving the animals’ health and animal products quality. Phytobiotics contain many bioactive compounds such as alkaloids, glycosides, tannins, saponins, essential oils, and, flavonoids, to which they owe their antioxidant, antibacterial, and anti-inflammatory properties [8,9,10]. Phytobiotic materials derive from those parts of plants in which the highest accumulation of active ingredients is found, that is, leaves, rhizomes, roots, flowers, bark, fruits, and/or seeds [11]. This paper aims to review scientific publications on the influence of herbal additives for broiler chicken diets on the fatty acid profile of poultry meat.

## 2. The Role of Poultry Meat in a Healthy Human Diet

Well-balanced diets containing different groups of foodstuffs, including fruit and vegetables, wholegrain cereals, and products of animal origin, are best for human health. Meat is particularly essential as a source of complete protein, vitamins—notably from the B group—and minerals (mainly iron). Fat is an important component in nutrition, supplying energy and fat-soluble vitamins such as vitamin E [5]. Despite these positive nutritional aspects, consumption of red meat is associated with a risk of various diseases that have been increasingly common in developed countries [12,13]. In turn, due to the good fatty acid profile and low content of cholesterol and fat, poultry meat is deemed a product having a dietary value [14]. It contains a considerable amount of PUFA, and a regular supply of these fatty acids is indispensable to ensure the correct functioning of the body, and—in addition—mitigates and can even prevent many civilization diseases such as cardiovascular disease and heart stroke or some autoimmune conditions and cancers [15]. Poultry meat has a high nutritional value, making it one of the most popular consumer choices [7]. Consumers’ awareness increases, and they want their diets composed of high-quality products.

On the one hand, evidence exists that consumption of long-chain fatty acids omega-3 (n-3) is beneficial in terms of nutrition, but on the other hand, variations in the fatty acid composition have a strong influence on the technological properties of meat such as tenderness, the content of intramuscular fat, shelf life and development of flavor during cooking. Moreover, a high level of PUFA in meat can lead to post-mortem oxidation of lipids, which may have a negative effect on the flavor and color of meat [16]. Even with antioxidant protection, lipids can still be oxidized to some extent. According to Faustman et al. [17], beef has a higher lipid oxidation capacity than pork and poultry meat.

From the point of view of human health, poultry meat is beneficial due to its low-fat content and a relatively high level of PUFA [1]. These acids are believed to be essential fatty acids, which means they need to be sourced from a diet (although some of them originate in a synthesis process, mainly in the liver) [18]. PUFA differs from SFA and MUFA by the presence of two or more carbon double bonds in a fatty acid chain [19]. Two main classes of PUFA are n-3 and omega-6 (n-6) fatty acids. Eicosanoids derived from n-6 acids are generally stronger mediators of inflammations, vasoconstriction, and platelets aggregation than eicosanoids derived from n-3 acids [20]. Therefore, with higher concentrations of n-3 EPA (eicosapentaenoic acid) and DHA (docosahexaenoic acid), and lower concentrations of arachidonic acid (C20:4 n-6), eicosanoids show less inflammatory activity [21]. Intake of the recommended amounts of PUFA, and particularly n-3 acids, is indispensable to ensure the correct functioning of the human body and essential for preventing and mitigating several diseases of civilization such as cardiovascular diseases, skin diseases, autoimmune conditions, and certain forms of cancer—breast, colon and prostate cancer [22,23,24,25]. EFSA [26] recommends a daily intake of EPA + DHA amounting to 250 mg, while the Institute of Medicine [27] refers to levels of α-linolenic acid (ALA), suggesting intake amounting to 1.1–1.6 g per day.

The two most common PUFAs in meat are linoleic acid (18:2 n-6) (LA) and ALA (18:3 n-3). Poultry meat rich in PUFA is particularly prone to lipid oxidation [28], which is a serious issue for the meat industry due to the aroma, taste, texture, and nutritional value of meat products [29]. In addition, certain compounds formed during the oxidation of lipids have an adverse effect on the consumer’s health due to their mutagenicity, carcinogenicity, and cytotoxicity [30].

Monounsaturated fatty acids (MUFA) derive partly from SFA synthesis and partly from a diet. The principal MUFA in meat is oleic acid (18:1 n-9), having cholesterol-lowering properties [18]. Next to PUFA, MUFA is also beneficial to health. Adequate amounts of MUFA (15–20% of the diet’s energy value) contribute to reducing the low-density lipoprotein (LDL) fraction of cholesterol and support body cells growth and maintenance [31].

The principal SFA in meat is palmitic acid (16:0) and stearic acid (18:0) [18]. Excessive intake of SFA is associated with raised LDL levels in the blood and exacerbated internal inflammatory conditions. Healthy adults should reduce SFA intake to not more than 10% of the diet’s energy value. Those with verified increased LDL cholesterol levels should decrease the intake of SFA to not more than 7% of the total calorie intake [32]. As the meat of slaughtered animals living on land is the main source of SFA [33], human health organizations often recommend replacing red meat with products richer in unsaturated fatty acids (UFA) (e.g., poultry or fish) [34]. Givens et al. [35] demonstrated that consumption of poultry meat contributes to increasing the supply of EPA and DHA in the diets of inhabitants of the United Kingdom. Out of their estimated daily intake of 244 mg, 26.4 mg derives from poultry, which corresponds to 72% of all meat. Such a high percentage is due to the large consumption of poultry (mainly chicken meat) compared to other types of meat and a relatively high percentage of n-3 PUFA in poultry meat.

## 3. Impact of Herbal Additives on the Fatty Acid Profile of Poultry Meat

The fatty acid profile of chicken meat is affected by the birds’ diet [4] and genetic factors [36]. Compared to other meat, chicken meat is richer in PUFA because the diet of broilers is generally rich in PUFA [37]. Recently, nutritional procedures to alter the fatty acid profile of meat have been a popular research topic. They are also the most practical way of manipulating the fatty acid profile in meat. Works discussing the effect of herbs beneficially modifying the fatty acid profile of chicken meat and eliminating negative effects of lipid oxidation in meat have gained increasing popularity [28,38]. It can be supposed that these are the antioxidant properties of flavonoids, carotenoids, essential oils, and other components of plants that may affect the fatty acid profile in tissue lipids and the oxidative stability of meat, as well as limit the deterioration of meat quality in storage. At present, the practices of enriching feed rations for birds with plant extracts have enjoyed great interest due to multiple beneficial uses of such extracts and, simultaneously, the possibility of increasing the production capacity and enhancing poultry health control [39,40]. Phytobiotics can improve the taste and smell of feed, increase its attractiveness and intake, regulate digestive functions, modify the microbiota of the digestive tract, and—consequently—contribute to weight gain and increased feed conversion rates, which is particularly significant given the increasing cost of feeding [41].

Herbal additives can be administered to birds as single herbs or mixes of different herbs—both fresh and dry or as extracts [42]. Herbs and herbal products contain antioxidants that can prevent the oxidation of UFA and cholesterol present in animal-origin products such as meat and eggs [43] and increase the antioxidant potential of the animal’s body [44,45,46]. Plants showing such properties include rosemary, salvia, oregano, thyme, and various extracts of the same [41]. This review of studies focuses on the use of various herbs, including Tilia cordata (small-leaved lime), Urtica dioica (common nettle), Humulus lupulus (common hop), Viola tricolor (heartsease), Melissa officinalis (lemon balm), Mentha piperita (peppermint), Achillea millefolium (common yarrow), Crataegus oxyacantha (hawthorn), Salvia officinalis (common sage), Camelina sativa (false flax), Centella asiatica (Asiatic pennywort), Curcuma longa (turmeric), Origanum vulgare (oregano), Eucalyptus globulus (blue gum), Sauropus androgynus (star gooseberry), Panax quinquefolius (Panax ginseng) and Pulicaria gnaphalodes (common fleabane), as feed additives for poultry featuring properties that modify the fatty acid profile of poultry meat, described in 25 original scientific papers from the past 15 years (Table 1). The works were sought in the PubMed and Google Scholar databases from November to December 2021. The search keywords were “herbs”, “fatty acid profile”, “phytobiotics”, “chicken”, and “poultry”—both in English and Polish. One hundred twenty-eight articles were found matching the topic.

### 3.1. Review of Studies

The presence of herbs in feed rations alters the fatty acid profile of both intramuscular fat and abdominal fat. Szkucik et al. [47] conducted an experiment in which chicks were allocated to experimental groups, each of them receiving feed with a 2% admixture of one of the following herbs: pansy, hop, lime, lemon balm, mint, or nettle. The results showed that the herbs had a significant influence on the fatty acid profile of the meat. An overall increase in SFA percentage in intramuscular fat was observed in all experimental groups, whereas all herbal additives used in the experiment significantly reduced the percentage of SFA in abdominal fat. The content of MUFA was comparable in all groups. Some differences were noted in the fatty acid profile of intramuscular fat after using mint, which increased the level of MUFA. Herbal additives had a significant effect on the profile of PUFA—lime and lemon balm showed a beneficial influence on the content of n-6 fatty acids in intramuscular fat. In abdominal fat, the amount of PUFA (both n-3 and n-6) decreased only after using hop, while other herbs increased it. The most desirable fatty acids ratio was found for feed with an admixture of mint and pansy—the percentage of PUFA increased, and simultaneously the level of SFA dropped. These results are confirmed by the research carried out earlier by Maślanko and Pisarski [60]. According to Winiarska-Mieczan et al. [70], the PUFA:SFA ratio in the muscles of broiler chickens ranges from 0.3 to 2.0, depending on the diet used and feed additives. The PUFA:SFA ratio has become a key parameter for evaluating food’s nutritional values and healthiness [71]. According to Simopoulos [72], the recommended PUFA:SFA ratio should be 1: 5. Since some types of meat naturally have an adverse PUFA:SFA ratio, their consumption can be a factor leading to an imbalance in fatty acids intake by present-day consumers. In the study by Eleroğlu et al. [53], the PUFA:SFA ratio in breast meat fluctuated from 3.47 to 2.78 and was improved after supplementation with oregano and lemon balm. In addition, those chickens showed higher levels of LA but lower levels of ALA compared to the control group.

Nettle is a perennial plant generating relatively low costs and is readily available in different parts of the world. Its beneficial impact on the fatty acid profile in intramuscular fat of pigs was demonstrated (reduced MUFA, increased PUFA) [73], but similar studies are conducted for broiler chickens. Dukić-Stojčić et al. [48] designed an experiment to examine the effect of fresh nettle supplementation on the fatty acid profile of chicken meat. In the group of chickens receiving 40–80 g of fresh nettle per day with their feed, the percentage of MUFA was significantly lower; in turn, the level of PUFA was significantly higher than in the no-herb group. Furthermore, fresh nettle raised the concentration of LA and ALA and reduced the n-6:n-3 fatty acids ratio in the breast muscle. The authors of the cited study noted an increase in the percentage of PUFA, LA, and ALA and a narrowing of the n-6:n-3 ratio in the breast muscles of Redbro chickens after supplementing their feed with fresh nettle. This is undoubtedly a result of the high content of bioactive components in nettle, including vitamins, organic acids, tannins, flavonoids, carotenoids, chlorophyll, xanthophyll, amines, mucilage, and wax [74]. The results of the Dukić-Stojčić et al. [48] partially differ from those obtained by Koreleski and Świątkiewicz [61]. Supplementation with sage caused an increase in n-3 fatty acids in the breast muscle of chickens compared to the control group. Supplementation of the diet with sage extract increased the levels of C18:0 and C20:0 but also decreased the level of C18:1 and C18:3 in comparison with the control group and chickens fed synthetic xanthophyll. The PUFA n-6:n-3 ratio was also lowered as a result of adding sage. These results may suggest an effect of the sage extract on fat metabolism. Skomorucha et al. [2] examined 640 broiler chickens to demonstrate that 2 mL of a common nettle extract per 1 L of water positively altered the fatty acid profile, mainly in broiler chickens’ leg muscles. Common nettle added to feed decreased the content of SFA (C10:0) and MUFA (C18:1) compared with the control and the lemon balm group and with the lemon balm and the lemon balm salvia group. In turn, both the chickens receiving lemon balm and those fed with salvia showed a decreased content of n-3 fatty acids compared to the control group.

Certain herbs, including their properly selected mixtures, can have a positive effect on the animal body. Marcinčákova et al. [49] examined the impact of feed with lemon balm (2%) and a combination of hawthorn (1%) and common yarrow (1%) on the fatty acid profile of chicken meat. In the breast meat of chickens supplemented with lemon balm, the percentage of MUFA was lower, and PUFA increased compared to the control group. In turn, in the group where a combination of hawthorn and yarrow was used, the percentage of SFA was higher, and that of PUFA was lower in comparison with the control group.

Feeding PUFA-rich components, particularly those containing n-3 acids (Camelina sativa oil or expeller), to broilers can be an effective way of improving both the health status of animals and the quality of their meat. According to Orczewska-Dudek and Pietras [50], broiler meat enriched with n-3 acids can make an alternative source of these fatty acids in the human diet. Control chicks were fed standard grower-finisher rations containing 60 g/kg rapeseed oil. Experimental components—Camelina sativa oil and an expeller—were included in a diet based on wheat and soy in the amount of 40 and 100 g/kg, respectively. The results of studies revealed that the amounts mentioned above of C. sativa oil and expeller introduced to the broilers’ diet considerably increased the share of n-3 PUFA, decreased the n-6:n-3 fatty acids ratio, and reduced the content of MUFA. The obtained results confirmed the data reported by Aziza et al. [62]. In this study, supplementing Camelina sativa led to significant increases in total n-3 fatty acids, C18:3 n-3, 20:5 n-3, 22:5 n-3, 22:6 n-3 both in the breast and in the tight muscles. Similar results were obtained by Thacker and Widyaratne [63] but for abdominal fat and by Ryhänen et al. [64] but only for C18:3 n-3 in the thigh muscle. Some researchers have also found that after supplementation with Camelina sativa, the concentration of 18:2 n-6 increases in both the breast and thigh muscles and in the abdominal fat [65]. The increase in the content of PUFA in meat contributes to lipid oxidation, which may affect the color, taste, and finally, oxidative stability of meat in storage [75].

Ramiah et al. [51] randomly assigned 240 broiler chickens to four groups: control, 0.5% powdered garlic, 0.5% Asiatic pennywort, and 0.002% virginiamycin. Chickens fed diets containing pennywort featured the highest level of ALA among all the experimental groups (*p* < 0.05). By contrast, diet showed no significant influence (*p* > 0.05) on the percentage of SFA and MUFA. Also, Haščík et al. [56] verified the effect of garlic and oregano combined with humic acids (80% humic acids + 20% garlic powder or 90% humic acids + 10% oregano powder) on the fatty acid profile in the breast and thigh meat of Ross 308 broiler chickens. The outcome of the above-quoted experiment corroborated that adding powdered garlic and oregano together with humic acids can have a beneficial effect on the fatty acid profile of chicken meat. In his doctoral dissertation, Wysmyk [41] presented the results of studies concerning the supplementation of broiler chicken diets with oregano oil. The preparation significantly altered the fatty acid composition and their totals both in breast and thigh muscles, in particular when mixed with wheat-based feed rations. It had the best influence on the n-6:n-3 fatty acids ratio when mixed with water (150 mL/L) and administered with wheat and barley feed containing 30% of barley.

The study of Galli et al. [52] presented the effect of feed supplementation with turmeric on the fatty acid profile of chicken meat. Two hundred twenty-five broiler chickens were allocated to five groups: negative control, positive control, 50 mg/kg turmeric with feed, 100 mg/kg phytogenic additive with feed, and a combination of 50 mg/kg turmeric and 100 mg/kg phytogenic. After 44 days of the experiment, the total SFA in meat was lower in all experimental and positive control than in the negative control groups. However, significantly higher levels of stearic acid (C18:0) were measured in the meat of all supplementation groups than in the negative control group. The situation was reversed for all PUFAs (C18:2 n-6, C18:3 n-3, C22:6 n-3), as their total was higher in all groups than in the negative control group. In turn, MUFA (C22:1 n-9, C16:1, C20:1 n-9) levels were higher in the test groups than in the negative control group. Curcumin and phytogenic microcapsules (carvacrol, thymolan, and cinnamaldehyde) can be successfully included in broiler feed. These additives improve meat quality, particularly by increasing the content of healthy PUFA. Hashemipour et al. [57] found that feed supplemented with a phytobiotic preparation containing an equal mixture of thymol and carvacrol in four doses (0, 60, 100, and 200 mg/kg of the diet) had a positive effect on the fatty acid profile of Ross 308 broiler chickens. In the above-quoted study, a decrease in total SFA was observed along with an increase in total PUFA and n-6 acids in thigh muscles compared to the control group receiving the standard feed.

Bluegum supplements can have a positive influence on the fatty acid profile in chicken thigh muscles [54]. The experiment involved 600 chicks that for 42 days were fed a basic diet containing corn and soy meal (control group) or a basic diet with an admixture of blue gum essential oil in different concentrations (250, 500, 750, and 1000 mg/kg). The results imply that the experimental medium linearly decreased the concentration of C14:0, C16:0, C18:0, and total SFA, while it linearly increased the content of C18:2, C18:3, C20:4, and total PUFA (*p* < 0.05). However, no material differences in the content of C20:0, C14:1, C16:1, C18:1, C20:1 fatty acids, and total MUFA in the thigh muscles of broiler chickens were identified between experimental groups. Among the four doses of blue gum, 1000 mg/kg performed best.

Shirani et al. [55] attempted to investigate the impact of common fleabane on the fatty acid profile of chickens’ breast and thigh muscles. The thigh muscles of chickens receiving probiotics and fleabane had a lower total content of SFA than those of chickens receiving antimicrobial growth promoters and of the control group (*p* < 0.05). Increased content of MUFA in thigh muscles was observed only for 0.2% and 0.3% of fleabane added to the feed compared to the control group and the antimicrobial growth promoter group (*p* < 0.05). A similar effect was observed for PUFA, but only when the dose of herbs was 0.3% (*p* < 0.05). All the experimental media, except the antimicrobial growth promoter, increased the content of n-3 fatty acids and the PUFA:SFA ratio in thigh muscles (*p* < 0.05). However, dietary procedures did not affect the fatty acids composition in breast muscle samples, which can be ascribed to the fact that thigh meat is fatter than breast meat. Similar results in the PUFA group were obtained for Lippia javanica by Mpofu et al. [66] for breast muscles but additionally reduced to an n-6:n-3 ratio in breast muscles. Zdanowska-Sasiadek et al. [67] received similar results, only for the Prisma Jet preparation consisting of tetterwort, bloodroot, plume poppy, and Kelway’s coral plume. In the meat of chickens for which herbs were added to the diet, decreased SFA (in breast muscle and abdominal fat), increased total MUFA (in abdominal fat), and promising results for PUFAs were noted—increased total PUFA (in breast muscle and abdominal fat), increased n-3 and n-6 (in thigh muscle and abdominal fat, and breast muscle and abdominal fat, respectively) and an increased n-6:n-3 ratio in both groups.

Santoso et al. [58] conducted research to evaluate the influence of medicinal herbs on fat deposition and the fatty acid profile in broiler chicken meat. Birds from experimental groups received 5% of powdered leaves of Sauropus androgynus, bay leaf, basil, papaya, or moringa with their feed. The experiment’s outcome shows that herb additives significantly increased the content of n-3 fatty acids and decreased the content of total fat, oleic acid, and omega-9 fatty acids (n-9).

By contrast, studies carried out by Chung and Choi [76] did not find any influence of red ginseng (expeller, fermented expeller, juice) on the fatty acid profile in the breast and thigh meat of Arbor Acres broilers. The above-quoted authors highlighted a need for continued research to identify the mechanisms underlying changes in the fatty acid profile of chicken meat. However, Lai et al. [59] demonstrated that ginseng included in broilers’ diet containing plant or animal fat at the grower stage could be a real strategy to control lipid oxidation in meat products stored in a refrigerator.

According to Gálik et al. [68], herbal supplementation of turkeys also changes the fatty acid profile of the meat. The use of oregano and anise in a dose of 1 kg/L t of the feed mixture resulted in a reduction of C20:4 n-6 in the thigh muscles, C18:1 MUFA in the breast muscles, as well as a decrease and increase in some SFA (C10:0, C12:0, C14:0 and C15:0, C17:0, C18:0, respectively). Contrary to these findings is the study by Hristakieva et al. [69] in which SFA, MUFA and PUFA content in both breast and thigh muscles did not demonstrate any significant differences between experimental groups (1% addition of the following herbs: common chamomile, rosemary, narrow-leaved lavender, origanum, thyme and St. John’s wort). As the content of C20:5 n-3 in breast meat was concerned, it was <0.001% in all studied groups.

**Figure 1:**
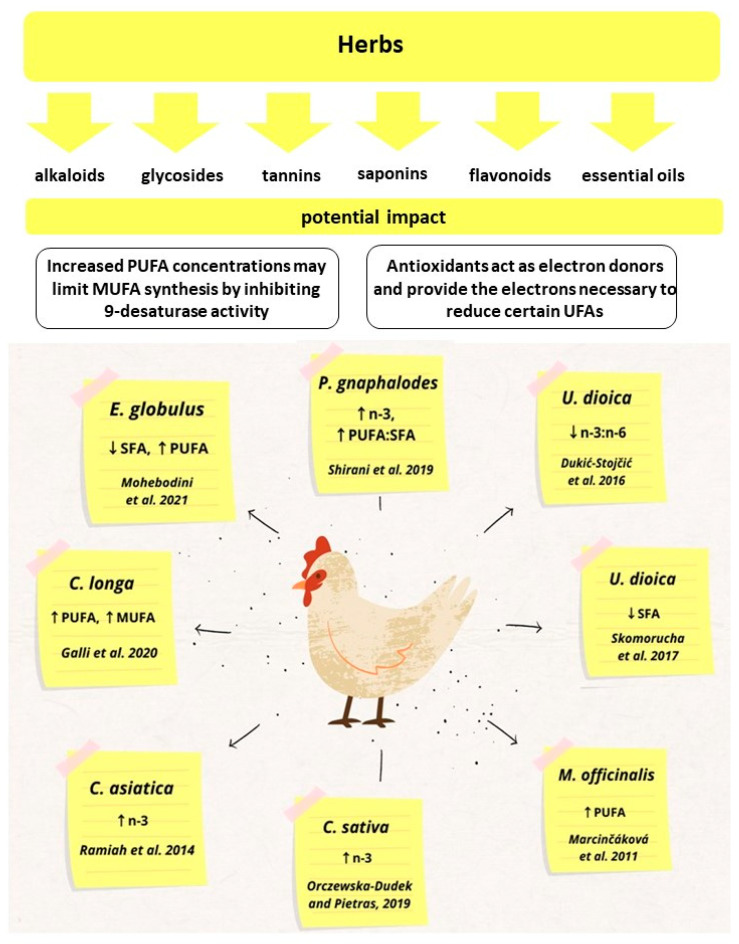
The positive effects of herbal additives on broiler chicken feed.

The positive effects of herbal additives on broiler chicken feed are presented in Figure 1.

### 3.2. Active Ingredients in Herbs Influencing Fat Metabolism

Herbs are useful in animal nutrition due to the presence and activity of various bioactive ingredients. These ingredients can show a biological impact on the body, including affecting fat metabolism. Thanks to antioxidant activity, active ingredients derived from herbs prevent the peroxidation of lipids [41], increase the concentration of high-density lipoproteins (HDL) and reduce the concentration of the LDL fraction [1]. Santoso et al. [58] claim that the fatty acid profile of feed directly influences the fatty acid profile in products of animal origin, including meat. Thus, medicinal herbs containing ALA (e.g., nettle, evening primrose) can increase the level of ALA in broiler chicken meat. Furthermore, herbs contain antioxidants that contribute to inhibiting autoxidation in meat [77]. In this process, oxygen attaches to fatty acids. This is a chain reaction, and, once triggered, it continues automatically, providing a continuous supply of free radicals that give rise to new reactions [22]. Enriching animal feed with herbs increases the antioxidant potential in meat. This prevents adverse reactions leading to fat rancidity and can be significant for consumers due to the longer storage shelf life of meat [22]. Some studies suggest that an increase in the PUFA:SFA and n-6:n-3 ratio in meat can be triggered by the protective role of exogenous antioxidants because they are electron donors and supply electrons necessary to reduce certain UFA so that they can be metabolized by microorganisms [78].

The share of fatty acids in meat is deemed an important measure of meat quality. Some authors found that enriching chicken diets with antioxidants is an excellent method of reducing the level of SFA and increasing the level of PUFA in slaughtered chicken meat, which had a direct impact on the sensory, physical, and chemical properties of meat [1,38,69]. It was also observed that the compounds were more efficient when added to the ration as a mixture than when added separately [79]. Kamboh and Zhu [1] observed that purified genistein (flavonoid from soya seeds) and hesperidin (flavonoid from citrus fruits) included in broilers’ diet positively changed the proportions of fatty acids in breast muscles (less SFA and more PUFA). The same applies to herbs—diets of broilers supplemented with thymol, tannic acid, and gallic acid decreased total cholesterol in the liver by about 12% and increased the content of PUFA in breast muscles by about 6% [80]. Jung et al. [81] also noted a decreased percentage of SFA and MUFA and an increased concentration of PUFA in the breast muscles of broilers due to supplementation of a mixture of polyphenols (gallic acid) with LA. The above-quoted authors concluded that an increased concentration of PUFA in poultry can reduce the synthesis of MUFA by inhibiting the activity of the complex of Δ9-desaturase—a key enzyme transforming SFA into MUFA—and supplementation of antioxidants is a primary strategy to reduce SFA and increase the share of PUFA in chicken meat. In turn, higher levels of LA in the meat of chickens receiving feed with herbs can be due to the presence of ALA in herbs [58]. Due to the high content of phenolic compounds, polyphenols inhibit cholesterol oxidation, have an anticholesterolemic effect, and prevent UFA oxidation [82]. Phenolic compounds can reduce the concentration of SFA [83]. It was demonstrated that phytogenic products included in broilers’ diet linearly increase lipase activity [57].

Antioxidants in herbs affect the chickens’ microbiome structure, for instance, by inhibiting the synthesis of DNA and proteins in the cells of microorganisms and creating complexes with sterols present in the membrane of pathogenic microorganisms, which leads to membrane damage and, consequently, the disintegration of the cells [75]. In turn, the microbiome indirectly affects carcass fattening and the fatty acid profile, mostly because it induces various biodegradation processes in the alimentary tract and stimulates digestion and absorption of nutrients, including PUFAs. The intestinal microbiome produces fatty acids such as conjugated linoleic acid (CLA). Many studies imply that CLA causes a significant increase in liver catalase activity in chickens, which can be associated with fat tissue reduction [84].

## 4. Changes in the Intestinal Microbiota and the Fatty Acid Profile of Chicken Meat

### 4.1. Impact of Antioxidants Contained in Herbs on the Intestinal Microbiota

Antioxidants contained in herbal extracts and oils—such as tannins, flavonoids, polysaccharides, and saponins—have a beneficial effect on the composition of the intestinal microbiota [85] and improve the epithelial structure [86,87,88,89], concurrently inhibiting the development of pathogenic bacteria and stimulating the growth of probiotic bacteria (Lactobacillus, Akkermansia muchiniphila, and Bacillus) [90,91,92,93]. Phytobiotics present in herbs show an antibacterial and antiviral effect and increase cell proliferation and tissue regeneration [94,95]. The study by Ali et al. [96] suggests that interactions between the mucous membrane and pathogenic bacteria or their toxins lead to oxidative stress destroying intestinal mucosa and oxidizing lipids. Therefore, strongly antioxidant phytobiotics in herbs such as phenolic compounds, saponins, alkaloids, and terpenoids help reduce reactive oxygen species and maintain a proper intestinal mucosa condition. Mice receiving feed with cinnamon essential oil featured higher diversity of the intestinal microbiota, a reduced count of Helicobacter and Bacteroides, and an increased count of Bacteroidales, as well as bacteria producing short-chain fatty acids (SCFA) [97]. Interestingly, in the quoted study, the correlation analysis showed that the level of the tolled-like receptor (TLR4) and TNF-α was positively correlated with the count of Helicobacter but inversely correlated with bacteria producing SCFA.

### 4.2. Microbiota and Fat Metabolism

Intestinal bacteria play an important role in regulating fat metabolism, also of broiler chickens [98]. Lipid metabolism through intestinal microbiota, and in particular saturation with acid fats, affects the accumulation of fat in tissues [99]. The authors of the above-quoted study demonstrated that the nutrition pattern is not the only reason for the accumulation of acid fats and that intestinal microbiota reinforces the production of SFA. Hussein and Selim [100] informed that the supplementation of diets with a multi-strain probiotic (Lactobacillus acidophilus, Bacillus subtilis, and Aspergillus oryzae) increased the PUFA, n-3 fatty acids ratio and the PUFA:SFA ratio and decreased the n-6:n-3 ratio in chicken meat. These scientists attribute one of the likely reasons for fatty acid profile modification to the positive effect of probiotics on the intestinal microbiota and a change in lipid metabolism.

In the process of bacterial fermentation, SCFA is formed. They have degraded in three locations: liver cells, muscles, and colon epithelial cells [101]. Intestinal epithelial cells absorb SCFA due to the diffusion of protonated acids and the exchange of anions [102]. Active transport of SCFA through the mucous membrane takes place through the monocarboxylate transporter 1 isoform (MCT-1) and the sodium-coupled monocarboxylate transporter 1 isoform (SMCT-1) [103]. Butyrate, as the basic source of energy for colonocytes, stimulates adipogenesis and accumulation of lipids, most likely through collecting glucose and de novo lipogenesis. It can also inhibit lipolysis, most likely on the GPR41-dependent path. It was demonstrated that adipocytes are capable of utilizing butyrate by increasing adiponectin expression to capture glucose and improve insulin sensitivity. The inhibition of lipolysis, stimulation of glucose capturing, and induction of triglyceride synthesis caused by butyrate suggest its potential role in preventing or reversing hyperglycemia and hyperlipidemia [104]. SCFA, as a product of bacterial metabolism, is in many ways responsible for maintaining homeostasis in the body, including in the intestinal microbiome [105].

The SCFA are known to activate the oxidation of fatty acids and inhibit de novo synthesis and lipolysis [106]. Thigh meat contains more lipids than breast meat, and the reduced content of lipids in thigh meat can be explained by SCFA regulating the balance between fatty acid synthesis, fatty acid oxidation, and lipolysis in the body [107].

Despite having many benefits for the host, intestinal microbiota can have a direct or indirect adverse effect in certain special conditions. Intestinal microbes reduce fat digestibility by deconjugating bile acids [108,109]. Bile acids and their salts are essential for the emulsification and absorption of fat in the intestines. The intestinal catabolism of bile salts induced by different microbiota reduces the absorption of lipids and produces toxins deteriorating the growth of animals [110].

### 4.3. Effect of Prebiotics Supplemented in Chicken Diets on the Fatty Acid Profile of Meat

Prebiotics are proposed as efficient ingredients of feed that stimulate the colonization of beneficial microbiota in chickens. A prebiotic is defined as an indigestible compound that, being metabolized by intestinal microorganisms, modulates intestinal microbiota’s composition and/or activity, thus ensuring a beneficial physiological effect on the host [111]. Only a few references describe the effect of prebiotics on the quality traits of broiler chicken meat [112,113]. However, none of them analyzed the fatty acid composition. Authors of some previously published studies demonstrated that prebiotics can alter lipid metabolism [114] and improve the PUFA:SFA ratio in chicken meat [115,116], which is beneficial to human life. The experiment by Tavaniello et al. [117] presented the effect of prebiotics on the quality traits of broiler chicken meat and, in particular, focused on the analysis of the fatty acid profile. Authors of the above-quoted study randomly allocated 1500 breeding eggs (Ross 308) to four groups and in each research group, injected in ovo 0.2 mL of the solution containing: 3.5 mg of transgalacto-oligosaccharides per embryo, 0.88 mg of Laminaria spp. extract per embryo, 1.9 mg of raffinose oligosaccharides per embryo, and in the control group—0.2 mL of the physiological saline solution. The chorioallantoic membrane of eggs is highly vascularized and allows transferring of prebiotics from the air chamber into the cardiovascular system and further to the developing intestine. The total content of SFA in the breast muscle was higher in the prebiotic groups than in the control group, but no statistically significant differences were identified between the three prebiotic groups. The total content of MUFA in breast meat specimens was lower in the prebiotic groups compared to the control group; this mainly concerned the content of oleic acid (C18:1 n9). The total content of PUFA was higher in all prebiotic groups than in the control group. The administration of prebiotics did not affect the concentration of linoleic acid (C18:2 n-6), while significant differences in other LC-PUFA n-6 were observed between the groups—the concentration of γ-linolenic acid (C18:3 n-6) was higher in all prebiotic groups. In contrast to n-6 PUFA, the total content of n-3 PUFA was about two times higher in the prebiotic groups than in the control group. The concentration of α-linolenic acid (C18:3 n-3) significantly increased after the administration of prebiotics in contrast to the eicosapentaenoic acid (C20:5 n-3), which had lower concentrations in the prebiotic groups compared to the control group. In addition to the higher level of PUFA and n-3 fatty acids, meat from the prebiotic groups also showed other ratios, such as the n-6:n-3 ratio, PUFA:SFA ratio, and index of thrombogenicity, that were beneficial to human health. Literature provides limited information on the impact of prebiotics on fatty acid pattern modification in poultry meat, which should be explored in future research.

## 5. Perspectives and Summary

Fatty acids derived from meat and modification of their composition will remain an important area of research concerning the impact of animal feeding on meat quality. This is due to the key role of fat in animal growth, nutritional value, and product quality. More and more often, n-3 PUFA is deemed a significant component of a diet. Enriching favorite meat with PUFA is a better way of supplying n-3 PUFA than relying exclusively on foods containing more PUFAs but are less preferred (e.g., oily fish). There has been an upward trend in the availability of meat with increased levels of n-3 PUFA, particularly chicken meat [7].

Increasing the content of UFA in poultry meat is beneficial from the point of view of its nutritional and dietary value, but on the other hand, meat-rich in PUFA is particularly susceptible to lipid oxidation, which is a serious issue for the meat industry due to the negative impact on its smell, taste, texture, and nutritional value [70]. Oxidation reactions occurring in meat and meat products are accelerated by grinding, thermal processing, salting, and refrigerating due to the joint activity of UFA and pro-oxidants such as non-heme iron [118,119]. In addition, certain compounds formed during the oxidation of lipids have a negative effect on the consumer’s health as they show mutagenic, carcinogenic, and cytotoxic effects [30]. Antioxidants in herbs promote the oxidative stability of poultry meat, as was demonstrated by many authors [38,57,120,121]. Herbs and herbal products contain antioxidants that can prevent the oxidation of UFA and cholesterol present in animal-origin products and increase the antioxidant potential of the animal’s body.

However, the primary problem is not the optimum dosage of herbs and forms of their administration to chickens since some studies indicate that their efficiency depends on the dose of active ingredients. Sohaib et al. [122] demonstrated that UFA synthesis decreased along with an increase in the level of quercetin and α-tocopherol in the dose. Feeding ducks with feed containing 1% of powdered chokeberries did not affect the fatty acid profile of their meat [123]. Also, in the studies of Forte et al. [124], a water-based extract of oregano added to the feed for Ross 308 broiler chickens (150 mg/kg feed) did not influence the percentage of fatty acids in breast meat. The lack of influence of herbal additives on the fatty acid profile in poultry meat could be due to both additive type and content of active ingredients and dosage, the more that some authors noted a linear relationship between the number of herbs and their efficiency [55,125]. However, excessive doses of active ingredients present in herbs can have an adverse effect on the birds by decreasing the intake of feed due to its intensive taste and/or smell (due to essential oils) and by having a negative impact on the functioning of the body. Some authors note that large doses of phenolic compounds can have adverse effects due to pro-oxidative activity [126]. Phenolic compounds also have a strong chelating effect on minerals, e.g., Ca^2+^ [127], which can lead to calcium deficiency in poultry. Studies involving rats showed that 10% of tannic acid added to feed significantly reduces the absorption of iron, which led to anemia [128].

Herbs and bioactive components of herbs added to chicken diets can improve the quality of broiler chicken meat by altering the content of fatty acids. Nutritionists often criticize meat for supplying excessive amounts of SFA in the diet and contributing to the development of cardiovascular diseases, some cancers, and type II diabetes [31,32]. By contrast, meat can be a significant source of n-3 PUFA, and the intake of these acids is now commonly evaluated as low. Meat would be healthier and better perceived if it supplied less SFA and more PUFA, particularly n-3 PUFA [18]. Recent studies have shown that the fatty acids composition of meat can be altered by modifying the diet during animal production to improve the conformity of meat with nutritional guidelines. Enriching animal diets with active ingredients derived from various herbs will remain popular since the nutrients become an integral part of meat structures and are most likely stable both during processing and distribution. Chicken meat’s fatty acid profile can be improved by modifying chickens’ microbiota. For example, this can be achieved when antioxidant herbs are added to chicken feed and after the supplementation of prebiotics modulating the intestinal microbiota’s composition and/or activity. For consumers, this may improve food quality without changing their eating habits.

## Figures and Tables

**Table 1:** Various herbs as feed additives for poultry featuring properties that modify the fatty acid profile of poultry meat.

Herb	SFA	MUFA	PUFA	Design	Time of Experiment	References
*Tilia cordata*	↑ total in IF ↓ total in AF	↓ C 16:1 in IF ↓ C 18:1 in IF ↑ C 18:1 in AF	↑ C 20:3 n-6 in IF ↑ C 20:4 n-6 in IF	Broiler chickens (*n* = 210) were divided into 6 experimental groups and fed with 2% additions of specific herbs—*T. cordata*, *U. dioica*, *H. lupulus. V. tricolor*, *M. officinalis*, *M. piperita*	42 days	[47]
*Urtica dioica*		↓ C 16:1 in AF ↑ C 20:1 in AF	↓ C 18:2 n-6 in IF ↓ C 20:2 n-6 in IF ↑ C 20:4 n-6 in IF ↑ C 18:2 n-6 in AF ↑ C 20:4 n-6 in AF			
*Humulus lupulus*		↓ C 16:1 in IF ↑ C 16:1 in AF	↓ C 18:2 n-6 in IF ↑ C 20:2 n-6 in IF ↑ C 20:3 n-6 in IF ↑ C 20:4 n-6 in IF ↓ C 18:2 n-6 in AF ↓ C 18:3 n-3 in AF ↓ C 20:2 n-6 in AF ↑ C 20:4 n-6 in AF			
*Viola tricolor*		↑ C 18:1 in AF ↓ C 16:1 in AF ↑ C 20:1 in AF	↓ C 20:2 n-6 in IF ↑ C 20:3 n-6 in IF			
*Melissa officinalis*		↓ C 16:1 in IF ↑ C 18:1 in AF ↑ C 20:1 in AF	↑ C 20:3 n-6 in IF ↑ C 20:4 n-6 in IF ↑ C 20:3 n-6 in AF			
*Mentha piperita*		↑ C 16:1 in IF ↑ C 18:1 in AF ↓ C 16:1 in AF ↑ C 20:1 in AF	↓ C 18:2 n-6 in IF ↓ C 20:2 n-6 in IF			
*Urtica dioica*		↓ C18:1 in BM	↑ C18:2 n-6 in BM ↑ C18:3 n-3 in BM	Redbro chickens (*n* = 400) with dietary supplementation of fresh *U. dioica* 40–80 g/chicken/d	63 days	[48]
*Melissa* *officinalis*		↓ C18:1 in TM and BM	↑ C18:2 n-6 in TM and BM ↑ C20:2 in TM and BM ↑ C20:3 in TM and BM ↑ C20:4 n-6 in TM and BM ↑ C20:5 n-3 in TM and BM ↑ C22:5 in TM and BM ↑ C22:6 n-3 in TM and BM ↓ n-6:n-3 ratio in BM	Broiler chickens (*n* = 90) Ross 308 with supplementation of *M. officinalis* and a combination of *C. oxyacantha* and *A. millefolium*	41 days	[49]
*Achillea millefolium* *Crataegus oxyacantha*	↑ C16:0 in TM		↓ C20:4 n-6 in TM			
*Melissa officinalis*			↓ n-3 in BM	Broiler chickens (*n* = 640) Ross 308 with supplementation of extracts: *M. officinalis, S. officinalis*, *U. dioica* in the amount of 2 mL/L of water	42 days	[2]
*Salvia officinalis*			↓ n-3 in BM			
*Urtica dioica*	↓ C:10 in TM	↓ C18:1 in TM	↑ DHA in TM ↑ C20:4 n-6 in TM			
*Camelina sativa*		↓ total in BM	↑ C18:3 n-3 in BM	Ross 308 broilers (*n* = 456) had a diet based on wheat and soybean with supplementation of *C. sativa* oil 40 g/kg and expeller 100 g/kg	42 days	[50]
*Centella asiatica*			↑ C18:3 n-3 in BM	Cobb 500 broiler chicks (*n* = 240) were randomly assigned into four treatments group: control, 0.5% garlic powder, 0.5% *C. asiatica* and 0.002% virginiamycin	42 days	[51]
*Curcuma longa*	↓ total in BM	↑ C22:1 n-9 in BM ↑ C20:1 n-9 in BM ↑ C16:1 in BM	↑ C18:2 n-6 in BM ↑ C18:3 n-3 in BM ↑ C22:6 n-3 in BM	Cobb 500 broiler chickens (*n* = 225) were divided into 5 groups: negative control feed, positive control, supplemented with 50 mg/kg of *C. longa*, 100 mg/kg phytogenic, a combination of both 50 mg/kg *C. longa* and 100 mg/kg phytogenic	44 days	[52]
*Origanum vulgare*			↑ C18:2 n-6 in BM ↓ C18:3 n-3 in BM	Chickens (*n* = 240) fed diets supplemented with *O. vulgare* 10 g/kg basal diets) or *M. officinalis* 10 g/kg basal diets under an organic housing system	98 days	[53]
*Melissa officinalis*						
*Eucalyptus globulus*	↓ C14:0 in TM ↓ C16:0 in TM ↓ C18:0 in TM		↑ C18:2 in TM ↑ C18:3 n-3 in TM ↑ C20:4 in TM	Ross 308 chicks (*n* = 600) were fed a corn-soybean meal-based basal diet or basal diet with different *E. globulus* essential oil concentrations, including 250, 500, 750, and 1000 mg/kg	42 days	[54]
*Pulicaria gnaphalodes*	↓ total in TM	↑ total in TM	↑ n-3 in TM ↑ total PUFA in TM	Ross 308 male broiler chicks (*n* = 576) were randomly assigned into 6 dietary treatments: control group, 0.1%/0.2%/0.3% *P. gnaphalodes* powder addition, 0.1% probiotic mixture addition and 0.05% bacitracin methylene disalicylate addition to basal diet	42 days	[55]
*Origanum vulgare*			↓ n-6:n-3 ratio	Flex broiler chickens (*n* = 180) received *O. vulgare* oil in the amount of 150 mL/L as an additive to drinking water when feeding feed mixtures containing various sets of cereals	42 days	[41]
*Allium sativum*	↓ total in TM	↑ total in TM		Ross 308 broiler chickens (*n* = 200) were randomly divided into 4 groups (*n* = 50): control group without supplementation; experiment group 2% humic acids; 80% humic acids and 20% garlic powder and 90% humic acids and 10% oregano powder	42 days	[56]
*Oreganum sativum*	↓ total in TM		↑ total in TM			
Thymol and carvacrol	↓ total in TM	↑ total in TM	↑ total in TM ↑ n-6	Ross 308 male broiler chicks (*n* = 240) received dietary supplementation of phytogenic product containing an equal mixture of thymol and carvacrol at 4 levels (0, 60, 100, and 200 mg/kg of diet)	42 days	[57]
*Sauropus androgynus*		↓ C18:1 n-9 in TM ↓ total n-9 in TM	↑ C18:3 n-3 in TM ↑ total n-3 in TM	Female broiler chickens (*n* = 168) were distributed into 7 groups, 4 replicates, each replicate consisted of 6 broiler chickens, as follows: control, fed a diet with 5% *Sauropus androgynus* leaf powder, fed a diet with 5% bay leaf powder, fed a diet with 5% basil leaf powder, fed a diet with 5% papaya leaf powder, fed a diet with 5% Moringa leaf powder, fed a diet with 5% noni fruit powder	20 days	[58]
*Panax quinquefolius*			↑ total PUFA	Broilers (*n* = 150) were fed with one of six different grower diets. Control broilers were fed with either a standard commercial animal fat-based or a vegetable-oil-based grower diet. Experimental diets were formulated to also include either 0.1% or 0.2% (w/w) of ginseng prong powder in both the animal fat- and vegetable-oil-based formulations, respectively	42 days	[59]
*Humulus lupulus*	↓ total in AF	↑ total in AF	↓ total in AF ↓ n-6:n-3 ratio	Broiler chickens (*n* = 210) were divided into 7 groups (1 control and 6 experimental groups) and fed with 2% additions of a specific herb—*H. lupulus*, *T. cordata*, *M. officinalis*, *V. tricolor*, *M. piperita*, *U. dioica*	42 days	[60]
*Tilia cordata*			↓ n-6:n-3 ratio			
*Melissa officinalis*						
*Viola tricolor*						
*Mentha piperita*						
*Urtica dioica*			↑ total in AF			
*Salvia officinalis*	↑ C18:0 in BM ↑ C20:0 in BM	↓ C18:1 in BM	↓ C18:3 n-3 in BM ↑ total n-3 in BM ↓ n-6:n-3 ratio	Cobb chickens (*n* = 320) were divided into 8 groups in 5 replicates and fed with 560 mg dry extracts: *Echinacea purpurea* or *Thymus vulgaris* or *Salvia officinalis* or with 20 mg of *Tagetes* sp.	42 days	[61]
*Camelina sativa*			↑ total n-3 in BM and TM ↑ C18:3 n-3 in BM and TM ↑ 20:5 n-3 in BM and TM ↑ 22:5 n-3 in BM and TM ↑ 22:6 n-3 in BM and TM	Cobb chickens (*n* = 160) were fed a corn- and soybean meal-based diet with added *Camelina sativa* at 0% (control), 2.5%, 5% and 10%	42 days	[62]
*Camelina sativa*			↑ total PUFA in AF ↑ total n-3 in AF ↑ total n-6 in AF ↓ n-6:n-3 ratio in AF	Ross 308 broiler chickens (*n* = 180) were divided into 6 groups. The control diet was based on wheat and soybean meal contained 15% canola meal and the experimental diets contained 3%, 6%, 9%, 12%, or 15% camelina meal	22 days	[63]
*Camelina sativa*			↑ 18:3 n-3 in TM	Ross 308 broiler chickens (*n* = 196) were randomly allocated to the 3 dietary treatments: diets contained 0, 5, or 10% *Camelina sativa* expeller	37 days	[64]
*Camelina sativa*			↑ 18:2 n-6 in BM, TM, and AF	Ross 308 broiler chickens (*n* = 90) were divided into 3 groups: supplemented with soybean oil, rapeseed oil, or camelina oil	35 days	[65]
*Lippia javanica*	↓ total in BM		↑ total in BM ↑ total n-3 in BM ↑ PUFA:SFA ratio in BM ↓ n-6:n-3 ratio in BM	Broiler chickens (*n* = 180) were divided into 4 groups: negative control, positive control, 5 g *L. javanica*/kg of feed, and 12 g of *L. javanica*/kg of feed	42 days	[66]
*Chelidonium maius* *Sanguinaria canadensis* *Macleaya cordata* *Macleaya microcarpa*	↓ total in BM and AF	↑ total in AF	↑ total in BM and AF ↑ total n-3 in TM and AF ↓ n-6:n-3 ratio ↑ total n-6 in BM and AF	Ross 308 broiler chickens (*n* = 38,000) were randomly allocated to the 2 treatments: the control group (without Prisma Jet) and the experimental group (with Prisma Jet which contains natural active substances originating from plants) at a dose of 1 kg/t of starter and grower, and 2 kg/t of finisher mixture	42 days	[67]
*Origanum vulgare* *Pimpinella anisum*	↓ C10:0 in TM ↓ C12:0 in TM and BM ↓ C14:0 in TM and BM ↑ C15:0 in TM and BM ↑ C17:0 in TM and BM ↑ C18:0 in TM and BM	↓ C18:1 in BM	↓ C20:4 n-6 in TM	Female hybrid XL turkeys (*n* = 300) were randomly divided into two groups: control (fed with standard complete feed mixtures for fattening) and experimental (standard diets were supplemented with a blend of essential oils from origanum, anise, and citrus fruits as well as prebiotic-rich fructooligosaccharides in dosage 1 kg/L t of feed mixture)	84 days	[68]
*Matricaria chamomilla*	no significant differences were observed	no significant differences were observed	no significant differences were observed	Female turkeys poults (*n* = 105) were randomly allocated to 7 treatment groups (one control and six experimental groups received basal diet plus 1% supplemented with dry herbs	126 days	[69]
*Rosmarinus officinalis*						
*Lavandula angustifolia*						
*Origanum vulgare*						
*Thymus vulgaris*						
*Hypericum perforatum*						

AF—abdominal fat; IF—intramuscular fat; TM—thigh muscle; BM—breast muscle; SFA—saturated fatty acids; MUFA—monounsaturated fatty acids; PUFA—polyunsaturated fatty acids.

## Data Availability

Not applicable.
